# A sero-survey of toxoplasmosis in farm and non-farm children from Wisconsin, United States, 1997–1999

**DOI:** 10.1186/1471-2458-13-837

**Published:** 2013-09-11

**Authors:** Claudia Muñoz-Zanzi, Jessica Williams-Nguyen, Edward A Belongia

**Affiliations:** 1 Division of Epidemiology and Community Health, School of Public Health, University of Minnesota, 1300 South Second St., Suite 300, 55454 Minneapolis, Minnesota, USA; 2 Marshfield Clinic Research Foundation, 1000 North Oak Ave. (ML2), 54449 Marshfield, Wisconsin, USA

**Keywords:** *Toxoplasma gondii*, Zoonosis, Children, Rural, Urban, Sero-survey, Prevalence

## Abstract

**Background:**

Toxoplasmosis is among the most widespread and prevalent zoonosis in the world. People can become infected through ingestion of oocysts shed by felids or of tissue cysts contained in meat from infected animals. Acute infection can result in a wide spectrum of consequences, including flu-like illness and retinitis, as well as congenital infection in pregnant women. Severe disease can occur, especially if people are immunocompromised. Frequency of human infection varies substantially by region due to ecological, social, and cultural factors. The most recent nationwide prevalence estimates in children from United States were 3.6% in 6–11 year olds and 5.8% in 12–19 year olds. Because of the limited knowledge of the occurrence of common zoonotic pathogens in children in the United States, the objective of this study was to estimate the sero-prevalence of *T. gondii*-specific antibodies in children from the Marshfield area in Wisconsin and to examine the association between sero-positivity and farm living.

**Methods:**

Banked sera from 342 Wisconsin children collected in 1997–1999, aged 2 to 18 years, were tested for *Toxoplasma gondii*-specific IgG antibodies using ELISA. Recorded information included age, sex, and whether the child resided on a farm. Impact of assay accuracy, sensitivity and specificity, on sero-prevalence was examined using Bayesian methods.

**Results:**

Observed prevalence of *T. gondii*-specific antibodies was 10.8% (37/347). Adjusting for sensitivity and specificity of the assays yielded a prevalence estimate of 8.0% (95% probability interval: 4% - 12.4%). Children living on a farm had a 5 times higher odds of *T. gondii*-specific antibodies than children not living on a farm (OR=5.08, 95% CI: 2.2 – 11.6).

**Conclusion:**

Results suggest that even in apparently low-risk populations, the true extent of the infection in children is significant. In this study population, children living on farms were differentially exposed, with earlier and higher infection risk than children not living on farms. Findings highlight the need to increase awareness about toxoplasmosis acquired early in life and to improve our understanding of the ecology of *T. gondii* in rural environments from developed and developing countries.

## Background

*Toxoplasma gondii* is a zoonotic pathogen that widely infects animals across the globe. Felids are the definitive host of *T. gondii* and shed the oocyst-stage of the parasite in their feces. Human and animal infection can occur from ingestion of oocysts present in the environment, as well as in contaminated water or food. Human infection can also occur after ingestion of tissue cysts (bradyzoites) present in meat from infected animals. Frequency of human infection varies substantially by region due to ecological, social, and cultural factors. In the Midwestern United States, age-adjusted sero-prevalence of antibodies to *T. gondii* was estimated to be 20.5% among those older than 12 years during the years 1988 to 1994. Nationally-pooled sero-prevalence estimates in children were 3.7% and 5.2% in those aged 1–5 years and 6–11 years, respectively, during the same time period [1]. While newer estimates for the Midwest are not available, nationwide prevalence estimates in children for the years 1999 to 2004 were 3.6% in 6–11 year olds and 5.8% in 12–19 year olds [2].

Under endemic conditions, acute disease associated with post-natal infection is sporadic and may range from mild disease to systemic illness including malaise, lymphadenopathy, encephalitis, and retinitis. Some individuals, especially the immunocompromised, can develop severe disease with increased fatality rate [3-6]. After initial infection, the parasite ultimately forms cysts in tissues, with a strong tropism for the central nervous system [7], and individuals remained infected for life [8]. While the long-term health effects of infection specifically during childhood are not well understood, individuals post-natally infected with toxoplasmosis are susceptible to future development of ocular disease [9]. Furthermore, potential links between toxoplasmosis and mental illness are under investigation [10,11]. Because of the limited knowledge of the status of common zoonotic pathogens in children in the United States, the objective of this pilot study was to estimate the sero-prevalence of the *T. gondii*-specific antibodies in children from the Marshfield area in Wisconsin, in particular, to examine the association between sero-positivity and farm living.

## Methods

### Study participants

This study took advantage of banked sera from 342 farm and non-farm children from the state of Wisconsin in the United States who were part of the Marshfield Epidemiological Study Area (MESA) and were participants of an unrelated study [12]. MESA is a 14 ZIP-code region in rural north-central Wisconsin, where nearly all residents receive medical care from the Marshfield Clinic regional network. Detailed information about the study area has been previously reported [13]. Study participants were enrolled from children who were having a routine clinic visit (well-child or other primary care visit) during the period September 1997 to August 1999. Limited demographic information was available for the purposes of this study, consisting of age, sex, and whether the child resided on a farm. Children were classified as farm residents if a parent reported that the child lived on a farm that raised animals or produced animal products. Serologic testing was performed on de-identified banked sera. Use of samples was authorized by the Marshfield Clinic Research Foundation according to guidelines of the Office of Human Research Protections for exempt de-identified samples. Protocol was also approved by the University of Minnesota Institutional Review Board (No. 0710M18084).

### Laboratory analysis

A total of 342 serum samples were tested for *T. gondii*-specific IgG antibodies using a commercial ELISA kit (MP Biomedicals, Orangeburg, New York). A positive result was indicative of prior exposure to *T. gondii*. Assays were carried out in duplicate according to manufacturer instructions including criteria for validity of each run and selection of cutoff for result classification. Optical density (OD) results were read at 450 nm. For the purpose of the analysis, results that fell into the category “suspect” were assumed to be negative.

### Data analysis

Data were tabulated to obtain overall and group-specific sero-prevalences and exact confidence intervals (C.I.) for proportions. Chi-squared or Fisher exact tests were used for comparison of proportions. Optical density values were compared by Student’s t-test. The association between demographic variables and sero-status was analyzed using logistic regression, while considering effect modification and confounding. Statistical significance was set at *P* < 0.05. The fit of the final logistic regression model was assessed using the Hosmer-Lemeshow test and C-statistic. Statistical analyses were carried out using SAS statistical software, Version 9.2 (Institute Inc., Cary, NC, USA).

In order to examine the impact of sensitivity (SE) and specificity (SP) of the assay, a posterior distribution of the adjusted prevalence (*AP*), and 95% probability interval (P.I.), was obtained from the observed prevalence (*OP*) based on the relationship $\mathrm{AP}=\frac{\left(\mathrm{OP}+\mathrm{SP}-1\right)}{\left(\mathrm{SE}+\mathrm{SP}-1\right)}$[14] and Bayesian analysis. Beta prior distributions for SE and SP of the test and of the prevalence of infection, denoting the current level of knowledge around each parameter, were elicited from expert opinion. An independent researcher with extensive experience in laboratory diagnosis of toxoplasmosis was contacted and asked to provide the level of certainty (90%, 95% or 99%) on a minimum value and a most likely value (mode) for each parameter. Priors from expert opinion were *beta*(130.7, 15.4) for SE (95% certainty that SE>85% and mode of 90%), *beta*(99.7, 6.2) for SP (95% certainty that SP>90% with mode of 95%) and *beta*(5.6, 42.6) for prevalence (95% certainty that prevalence <20% with mode of 10%). Impact of the choice of SE and SP priors on *AP* was examined by repeating the analysis with different *beta* priors representing various modes, while keeping the same range of possible values. Uninformative *uniform* priors, which assumed that all values within the stipulated range were equally likely, were also used. A range between 85% and 100% was assumed for SE and between 90% and 100% for SP. Bayesian estimation of *AP* was carried out with the program WinBUGS, Version 1.4.3 (Imperial College and MRC, UK).

## Results

Age of children tested ranged from 2 to 18 years with a mean of 10.2 years. Fifty-four percent (183/342) were male and 46.2% (158/342) lived on a farm (Table 1). Overall *OP* was 10.8% (95% C.I.: 7.7% – 14.6%). Mean age of children who tested positive for exposure to *T. gondii* was 11.4 years with a range from 4 to 17 years. The average ELISA OD was significantly higher (*P* < 0.001) in children living on a farm (Mean: 0.57, SD: 0.79) compared to those who did not (Mean: 0.29, SD: 0.47). Similarly, in the univariable analysis, there was a statistically significant difference in sero-prevalence between children who lived on farms compared to those who did not (P < 0.001). There was no difference by sex (P = 0.230) or age (P = 0.145); however, there was a statistically significant increasing trend in sero-prevalence by age category (P = 0.049) (Table 1).

**Table 1:** Toxoplasmosis exposure by demographic characteristics in children from Wisconsin, United States 1997–1999 based on detection of pathogen-specific IgG antibodies

**Factors**	**No. positive**	**Sero-prevalence (95% C.I.)**^**a**^	**P-value**
All participants	37/342	10.8% (7.7 – 14.6)	--
Farm residence			<0.001
Yes	29/158	18.4% (12.7 – 25.3)	
No	8/184	4.4% (1.9 – 8.4)	
Age (years)			0.145, 0.049^b^
2–5	4/75	5.3% (1.5 – 13.1)	
6–10	9/91	9.9% (4.6 – 18.0)	
>10	24/176	13.6% (8.9 – 19.6)	
Sex			0.230
Male	23/183	12.6% (8.1 – 18.3)	
Female	14/159	8.8% (4.9 – 14.3)	

^a^Observed prevalence and 95% Exact binomial confidence interval.

^b^Chi-square test for trend.

Farm residence continued to be a strong predictor of sero-positivity after controlling for age in the multivariable model. Children living on a farm had 5 times the odds of having antibodies against *T. gondii* compared to children who did not live on a farm (OR = 5.08, 95% CI: 2.2 – 11.6, *P* < 0.001). In addition, odds of prior exposure to *T. gondii* was significantly associated with age as quadratic function after controlling for farm status (*P* = 0.042 for age, *P* = 0.068 for age^2^). At 5 years of age, a one year increase in age was associated with a 33% higher odds of having antibodies against *T. gondii* , while at 10 years of age the same increment was associated with a 6% higher odds. At 15 years of age, a one year increase in age was associated with a 14% decrease in odds of antibodies against *T. gondii*. Sex was not associated with previous infection with *T. gondii* (*P* = 0.356) and was removed from the final logistic regression model containing farm and age. No interactions were found to be statistically significant. Goodness of fit tests determined the appropriateness of the final model (Hosmer-Lemeshow test *P* = 0.230, c-statistic = 0.739).

**Figure 1:**
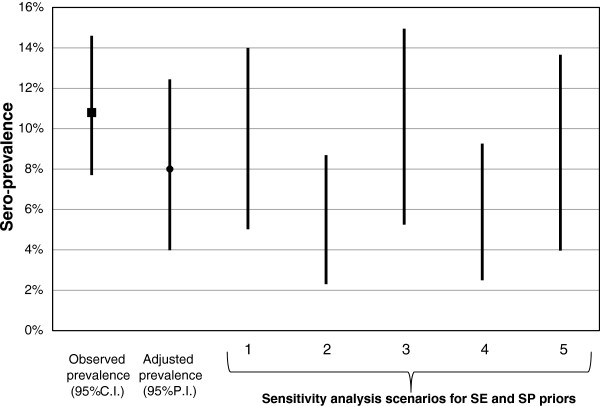
**Sensitivity analysis of the choice of priors for assay sensitivity (SE) and specificity (SP) on the Bayesian adjusted estimate of toxoplasmosis sero-prevalence in a population of children from Wisconsin, United States, 1997–1999.** Sensitivity analysis included various scenarios for most likely value while keeping the range provided by expert opinion. 1: SE >85% and mode = 100%, SP >90% and mode = 100%; 2: SE >85% and mode = 100%, SP >90% and mode = 91%; 3: SE >85% and mode = 86%, SP >90% and mode = 100%, 4: SE > 85% and mode = 86%, SP >90% and mode = 91%; 5: SE 85% - 100%, SP 90% - 100%.

After adjusting for SE and SP of the assay, the estimate of the overall sero-prevalence changed to 8.0% (95% P.I.: 4.0 – 12.4%). Modification of priors revealed some changes in the estimates of *AP*; however, overall, *AP* values were consistently lower than the *OP* and the various 95% P.I.s tended to overlap (Figure 1). The sensitivity analysis allowed us to investigate the impact of potential misclassification on the prevalence estimates, where for example, in Scenario 2 of high SE (mode = 100%) and low SP (mode = 91%), *AP* decreased to 5% (95% P.I.: 2.3% - 8.7%) to account for the large fraction of potentially false positive results (Figure 1). Conversely, Scenario 3 showed the higher *AP* (10.4%, 95% P.I. = 5.3% - 15.0%) that would result if assay SE was low (mode = 86%) and SP was high (mode = 100) due to adjustment for false negative results. The most conservative scenario, based on the uniform distributions, showed that the prevalence could be as low as 4% or as high as 13.7% (Scenario 5, Figure 1).

## Discussion

This pilot study demonstrated evidence of exposure to *T. gondii* in a population of children from the Midwest of the United States. Results revealed exposure as early as 2–5 years of age and highlighted a significant differential risk associated with farm vs. non-farm living. Toxoplasmosis is an established zoonotic infection in the United States and large-scale prevalence studies have documented its occurrence and distribution*.* The observed sero-prevalence of *T. gondii*-specific antibodies in non-farm children in this study was 4.4% (Table 1), which is in general agreement with the national estimates of 3.7 % to 5.8% for children in various age groups [1,2]. However, farm residence was found to be associated with a 5 times higher odds of sero-positivity. The study took advantage of existing banked sera from children; therefore, limited study participant data were available to make inferences about risk factors or sources of infection beyond residence. Nevertheless, results suggest that farm-related routes of exposure may play an important role for *T. gondii* transmission to children.

Post-natal transmission of *T. gondii* could have occurred by two main routes, exposure to oocysts shed into the environment by felids, including domestic cats, or the consumption of tissue cysts found in undercooked meat from infected livestock. Risk of exposure from these two sources is dependent on environmental factors and human behaviors and choices. Studies indicate that the risk of infection from meat consumption in the United States varies by type of meat and origin. Evidence of exposure can be high in pigs, chicken, and sheep raised outdoors; however, prevalence is commonly low in livestock raised in conventional high-biosecurity facilities [15,16]*.* Cattle are not an important carrier of *T. gondii*. Furthermore, inactivation of tissue cysts by meat processing (salt treatments), storage practices (freezing), and proper cooking (in particular pig and poultry) contribute to reduction in infection risk from meat consumption [16]. On the other hand, the large number of oocysts shed by cats and their long survival (up to 18 months under proper conditions) contribute to the potential for widespread environmental contamination [17], as well as the unrecognized ingestion of oocysts by people [18-20]. Congenital transmission cannot be ruled out as a source of infection in the sero-positive children found in this study; however, based on a reported congenital infection rate of 1 in 10,000 newborns [21], the likelihood that congenital infection could explain a significant fraction of the observed prevalence is unlikely. The higher risk of infection in children living on farms is consistent with previous reports [22,23]. Although not directly comparable, such reports suggested or hypothesized that environmental sources of infection seem to be significant in the studied age groups [22,24]; however, they also highlight the difficulties in attempting to identify specific sources from epidemiological data. The actual sources of infection in these young children remain to be investigated, revealing a gap of our understanding of the ecology of the pathogen in farm environments and of the level of awareness about infection risk by parents.

It is also important to note that although congenital toxoplasmosis is the subject of a significant amount of research, primary infection during childhood has received little attention. In this study, 7.8% of the children were already sero-positive by 10 years of age suggesting a potentially important level of exposure during early childhood. Studies of early exposure to *T. gondii* in high risk countries have found even higher sero-prevalences at these early ages, including a study in Panama reporting a prevalence of 49% by 10 years of age [25]. The significance of early infection in a still developing brain [26] by a pathogen with tropism for the CNS has not been examined. Furthermore, because of the differentially higher risk of infection on farms, it would be important to investigate the current incidence of infection in women of child-bearing age living on these farms areas and examine the status of public health prevention programs targeting rural populations.

As it is often done in prevalence studies, the reported *OP* estimate was the result of applying an imperfect laboratory assay (SE and SP are not 100%). Adjusting the *OP* estimates for the SE and SP of the assay, including the uncertainty about their real values, allowed us to provide a range within which the actual prevalence would likely fall. The *AP* estimates was slightly lower than the *OP*; however, these results still suggest that, even in apparently low-risk populations, the true extent of this common zoonotic infection is often under-estimated. It is important to emphasize that the study was done on sera collected several years ago in a specific group of children and results may not reflect current conditions. It would be suitable to investigate if results would be similar today, and in a larger population, considering changes in food-consumption habits (e.g. higher demand for locally-produced meat or from animals raised outdoors in lower biosecurity production systems) and in disease prevention campaigns.

## Conclusions

Children from the Midwest of the United States showed evidence of infection to *T. gondii* early during childhood. Sero-prevalence was significantly higher in children who lived on farms than in children who did not. Study findings underscore the need to improve our understanding of the potential impact of early infections in child development and to evaluate the efficacy of current zoonotic disease prevention programs. Pediatricians and public health officials should be aware of toxoplasmosis in children, in particular from rural areas.

## Competing interests

The authors declare that they have no competing interests.

## Authors’ contributions

CMZ contributed to the conception, design, laboratory analysis, data analysis, and writing of the manuscript. JWN contributed to data analysis and writing of manuscript. EAB contributed to sample acquisition and manuscript writing. All authors have read and approved the final manuscript.

## Pre-publication history

The pre-publication history for this paper can be accessed here:

http://www.biomedcentral.com/1471-2458/13/837/prepub
